# Evaluation and Comparison of Changes in Microhardness of Primary and Permanent Enamel on Exposure to Acidic Center-filled Chewing Gum: An *in vitro *Study

**DOI:** 10.5005/jp-journals-10005-1228

**Published:** 2014-04-26

**Authors:** Vijaya Lakshmi Mudumba, Radhika Muppa, NCH Srinivas, Duddu Mahesh Kumar

**Affiliations:** Senior Lecturer, Department of Pedodontics and Preventive Dentistry Narayana Dental College and Hospital, Nellore, Andhra, Pradesh India; Head, Department of Pedodontics, Panineeya Dental College Hyderabad, Andhra Pradesh, India; Reader, Department of Pedodontics, Panineeya Dental College Hyderabad, Andhra Pradesh, India; Reader, Department of Pedodontics, Panineeya Dental College Hyderabad, Andhra Pradesh, India

**Keywords:** Microhardness, Dental erosion, Chewing gum

## Abstract

**Objectives: **The study is to evaluate changes in microhardness of enamel after exposure to acidic center filled chewing gum on primary and permanent teeth.

**Methods: **Thirty primary and 30 permanent molar extracted teeth were painted with acid resistant varnish except a small window over buccal surface. Teeth were divided into four groups according to type of teeth and type of chewing gum (Center fresh and Bubbaloo) (D_1_, P_1_, D_2_ and P_2_); each tooth was exposed to whole chewing gum mashed with 5 ml of artificial saliva for five minutes at room temperature twice a day for 5 days. After the exposure, teeth were stored in deionized water and submitted for microhardness tests.

**Results: **Paired t-test and independent sample t-test were used for statistical analysis. A significant reduction in microhardness was found between exposed and unexposed areas in all groups. There was no statistically significant difference in reduction of microhardness to chewing gums, and between primary and permanent enamel.

**Conclusion: **There is a definite reduction in microhardness in all groups exposed to chewing gums. Both the chewing gums are equally erosive; both permanent and primary teeth were affected.

**How to cite this article: **Mudumba VL, Muppa R, Srinivas NCH, Kumar DM. Evaluation and Comparison of Changes in Microhardness of Primary and Permanent Enamel on Exposure to Acidic Center-filled Chewing Gum: An *in vitro *Study. Int J Clin Pediatr Dent 2014;7(1):24-29.

## INTRODUCTION

Prevalence of dental caries in most developed countries has declined with an increase in prevalence of other dental disorders, such as dental erosion.^1^ Changed dietary habits is one of the consequences of a modern life style which have to be taken into account when considering the augmented dental erosion status.

The dental erosion has become a major dental problem in both children and adults. It is defined as the loss of tooth substance by chemical processes (acids) not involving bacteria (Zipkin and McClure, 1949).^2^ In the incipient phase, enamel is dissolved without clinically detectable softening and dentin is affected only at a later point.^3^

Dental erosion is caused by a variety of extrinsic and intrinsic factors. Among the extrinsic factors is excessive consumption of acidic food stuffs as well as professional exposure to acidic environments where as chronic gastro intestinal disorders, anorexia and bulimia nervosa with fre­quent vomiting are considered the most frequent intrinsic reasons.^4^ pH of a dietary substance alone is not predictive of its potential as other factors modify the erosive process. These factors are chemical (pKa values, adhesion and che-lating properties, Calcium, phosphate and fluoride content) behavioral (eating and drinking habits, life style, excessive consumption of acids) and biological (fow rate, buffering capacity, composition of saliva, pellicle formation, tooth composition, dental and soft tissue anatomy).^5^

Regarding dental substrates, primary enamel and dentin are thinner than permanent. Erosive process reaches dentin earlier in primary enamel when compared to permanent enamel and this difference in susceptibility to erosion might increase over time and / or with decreasing pH of the acid.^1^ Progression of erosion correlates with age (erosion and wedge-shaped defects), consumption of dietary acids (erosion) and frequency of tooth brushing (wedge-shaped defects).^3^

Despite the advantages of chewing gums, a delivery vehicle for substances such as calcium, bicarbonate, carbamide, chlorhexidine, fluoride and xylitol to improve oral health and reduce caries,^6^ they can cause detrimental effects ranging from erosion to gastrointestinal disturbances.

This study was done to evaluate the changes in the micro hardness of primary and permanent enamel after exposure to two chewing gums as a whole.

## METHODS

The present study was carried out in the Department of Pedo-dontics and Preventive Dentisty, PMVIDS, Hyderabad in collaboration with DMRL (Defense Metallurgical Research Laboratory), Hyderabad.

All the primary and permanent extracted teeth were washed thoroughly under running tap water to remove blood, saliva, debris and cleaned with slurry of pumice. Then the teeth were examined under stereomicroscope to rule out presence of cracks and defects. Sixty primary and permanent teeth free of cracks and defects selected for the study were stored in deionized water till the experiment was started. All the surfaces of the teeth were painted with acid resistant paint except a small window over buccal surface. Groups are divided according to type of dentition (D — for Primary, P – permanent), chewing gum (1 — for Center fresh, 2 — for Bubbaloo). Groups were accordingly named as D_1_, D_2_, P_1_, P_2_ and each comprised of 15 teeth.

Artificial saliva was prepared in the department of biochemistry using 2 gm of methyl-p-hydroxy benzoate, 10 gm of sodium carboxymethylcellulose, 0.625 gm of KCl, 0.059 gm of MgCl_2_6H_2_O, 0.166 gm CaCl_2_2H_2_O, 0.804 gm of K_2_HPO_4_, 0.326 gm of KH_2_PO_4_ were measured with common balance and added to 1 liter of distilled water. Fluoride of 0.022 ppm was added to this solution. pH was checked with electronic digital meter and was 6.75.^7^

**Fig. 1:**
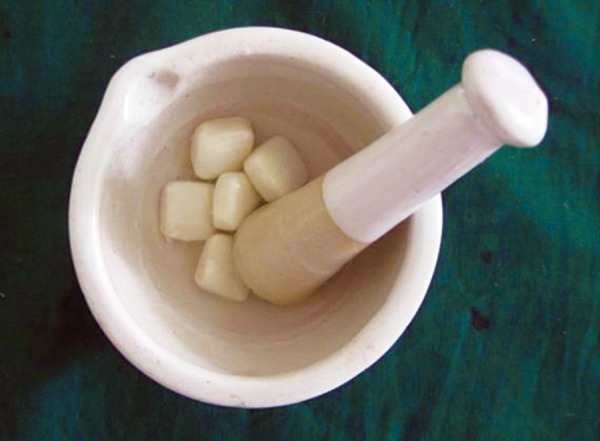
Five chewing gums with 25 ml of artificial saliva (5 ml of artificial saliva/chewing gum)

**Fig. 2:**
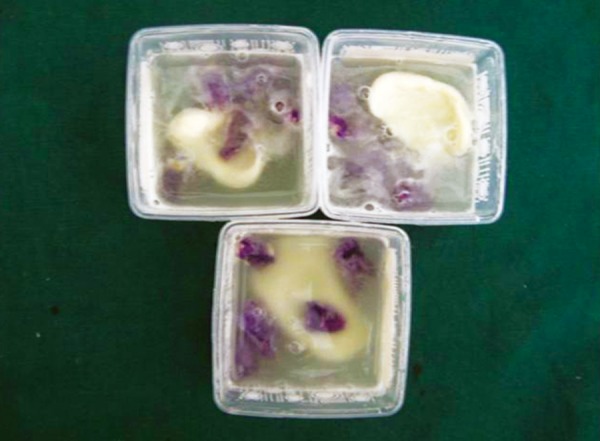
Exposure of teeth to whole chewing gum (center fresh) mixture (chewing gum mashed with artificial saliva)

**Fig. 3:**
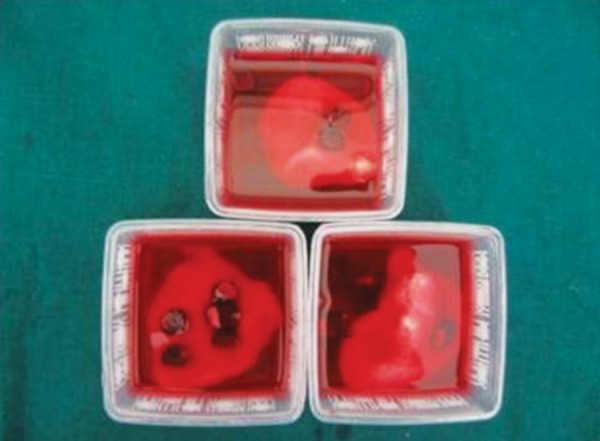
Exposure of teeth to whole chewing gum (Bubbaloo) mixture (chewing gum mashed with artificial saliva)

**Fig. 4:**
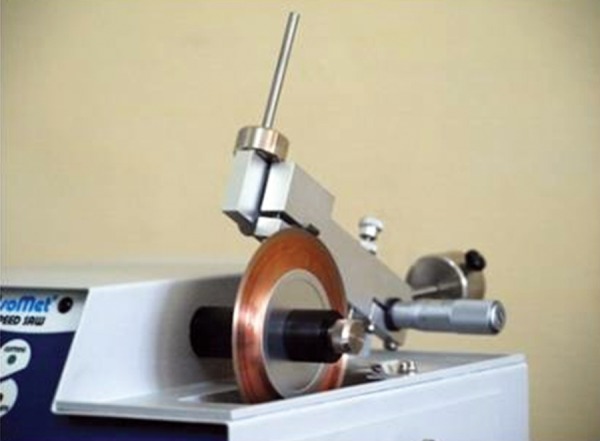
Isomet slow speed saw (Beuhler)

**Fig. 5:**
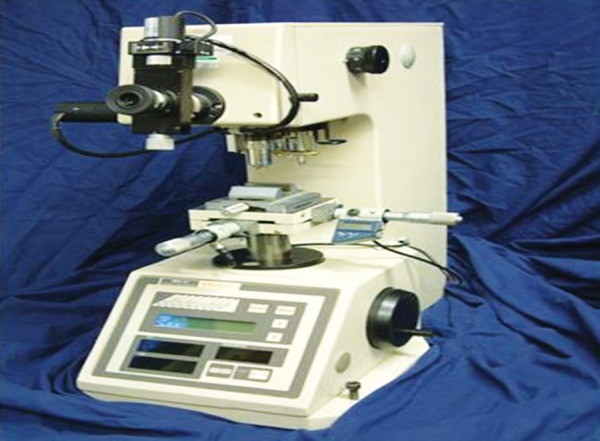
Knoop microhardness indenter

**Fig. 6:**
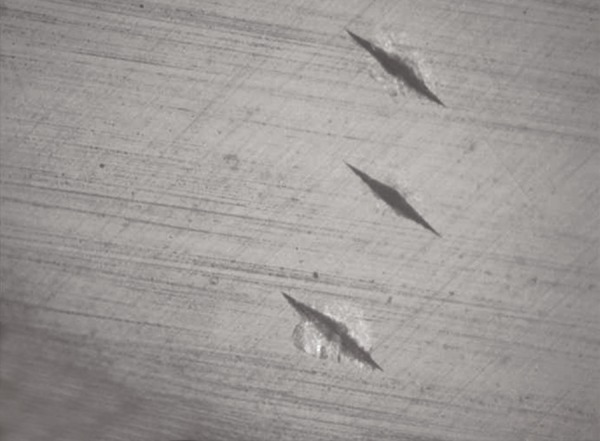
Three knoop microhardness indentations

Whole chewing gum was mashed together with 5 ml of artificial saliva using mortar and pestle (Fig. 1). The resultant liquid was used in the study during acidic exposure. Acid exposures of all four groups were done for 5 minutes at room temperature twice a day at 10 am and 1 pm for 5 days (Figs 2 and 3). After each exposure specimens were washed in deionized water for 20 seconds and immersed in artificial saliva at 37°C until the next experimental step. Artificial saliva was changed daily. After exposure, sectioning of specimens was done buccolingually through the window with an Isomet Slow speed saw (Beuhler) (Fig. 4). Specimens were subjected to microhardness tests with knoop diamond indentor (Fig. 5) with 50 gm load for 10 seconds at exposed and unexposed areas after mounting all the specimens in cold cure acrylic with cut surface exposed. Microhardness was tested at subsurface area (Fig. 6).

**Graph 1:**
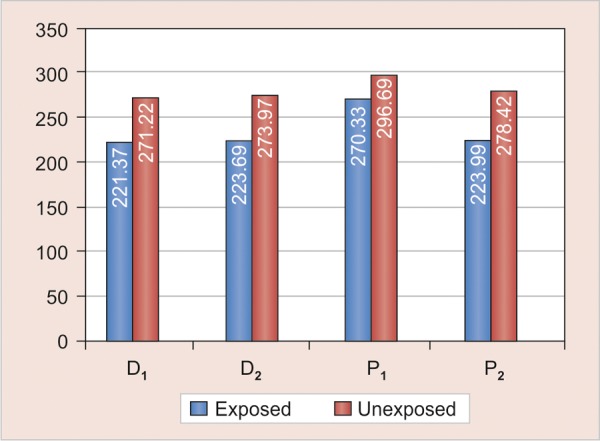
Comparison of means of knoop microhardness values between exposed and unexposed areas in each group

**Graph 2:**
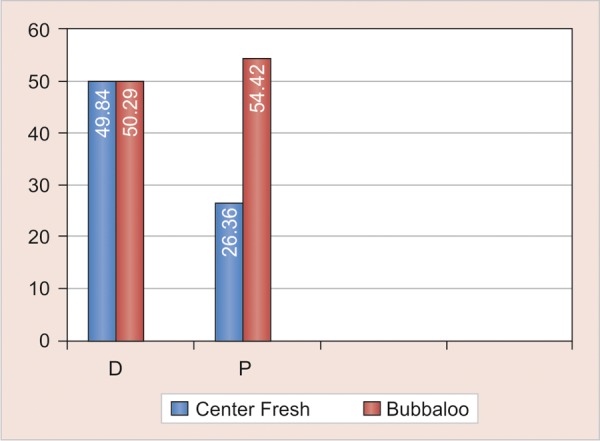
Comparison of difference in mean knoop microhardness values of unexposed and exposed areas exposed to center fresh and bubbaloo chewing gums

The results were tabulated and subjected to statistical analysis using Microsoft Excel software.

## RESULTS

**Graph 3:**
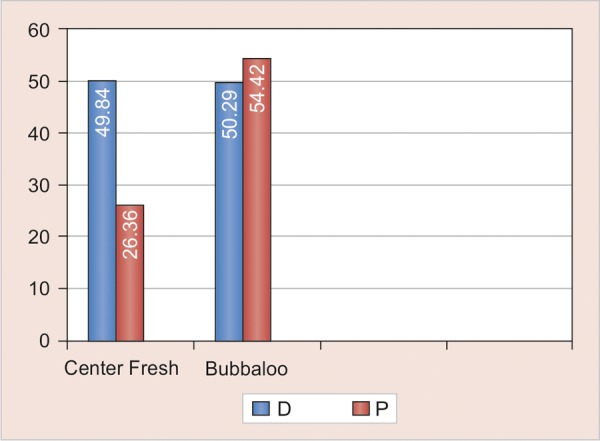
Comparison of difference in mean knoop microhardness values of unexposed and exposed areas between deciduous and permanent teeth

All experimental groups exposed showed significant reduction in microhardness (p-values of D_1_ = <0.001, D_2_ = 0.005, P_1_ = 0.015, P_2_ = 0.001) (Table 1) (Graph 1). There is no statistically significant difference in the reduction of microhardness with both types of chewing gums 1 and 2. (p values of comparison between D_1_ and D_2_ = 0.98, P_1_ and P_2_ 0.087) (Table 2) (Graph 2). There was no statistically significant difference in the reduction of microhardness between primary and permanent enamel (p-values of comparison between D_1 _and P_1_ = 0.835, D_2_ and P_2_ = 0.835) (Table 3 and Graph 3).

## DISCUSSION

Though dental caries is the most common dental health problem, other dental lesions such as dental erosion are becoming increasingly important. It has been a neglected problem because of unawareness of their causative factors and lack of immediate severe morbidity. Increase in the consumption of soft drinks and chewing gums have led to the augmented prevalence of erosion. Acidic center filled chewing gums have proven to be erosive^8^ in nature and are being used by children more frequently. In our study, we have selected two most commonly used center filled chewing gums, Center Fresh from Perfetti Van Melle and Bubbaloo from Cadbury Adams Pvt Ltd, and evaluated and compared their erosive effect on primary and permanent enamel *in vitro*.

Deionized water has been used in many studies as storage medium for extracted teeth.^9^ In the present study, the teeth were stored in deionized water after washing in running water, till experiment was started so that no change in the hardness of enamel is seen. Chewing gum was exposed to acid resistant varnish uncovered area (window) for 5 days, twice a day. A demineralization treatment of 5 minutes is representative of the effects of acidic beverage consumption. Although longer acid exposure times have been reported in the range of 10 to 60 minutes and shorter acid exposure times in the range of 1 to 4 minutes, it was considered that a 5 minutes exposure time would give an overall appropriate level of *in vitro *erosion severity.^10^ In this study, we have exposed the specimens to the contents of mashed chewing gum with artificial saliva for 5 minutes. As it is known that repeated application of demineralization cycle leads to a more severe damage of enamel apatite, which cannot be recovered even after an exposure to remineralizing solutions for several days. In the literature, studies have shown that acidic exposure for 5 days caused erosive effect.^11^ In this study, we have exposed buccal window of enamel to the contents of chewing gum for 5 days twice a day.

In this study, artificial saliva is used to simulate oral conditions and to compare the effect of chewing gum on teeth *in vivo*. As the amount of stimulated saliva secreted per minute is 1 ml/minute, and considering that child chews chewing gum approximately for 5 minutes, whole chewing gum mixture was prepared by mashing one chewing gum with 5 ml of artificial saliva, in the preparation of whole chewing gum mixture. The specimens were stored in artificial saliva after exposing the teeth to chewing gums. Artificial saliva was changed every 24 hours. After the completion of 5 day experimental procedure all specimens were removed from artificial saliva and stored in deionized water to prevent remineralization by artificial saliva of demineralized enamel.^12^

**Table 1:** **Table 1: **Comparison between means of knoop microhardness of exposed and unexposed surfaces in each group (n = 15) (paired t-test)

*Group*		*Hardness*		*Mean*		*SD*		*p-value*	
D_1_ deciduous teeth-Center Fresh		Exposed		221.37		20.72		<0.001			
		Unexposed		271.22		31.50					
D_2_ deciduous teeth-Bubbaloo		Exposed		223.69		41.21		0.005			
		Unexposed		273.97		48.94					
P_1_ permanent teeth-Center Fresh		Exposed		270.33		36.82		0.015			
		Unexposed		296.69		20.62					
P_2_ permanent teeth-Bubbaloo		Exposed		223.99		68.51		0.001			
		Unexposed		278.42		38.24			

**Table 2:** **Table 2: **Comparison of means of knoop microhardness values of unexposed and exposed primary and permanent teeth to 2 chewing gums center fresh and bubbaloo (n = 15)

*Dentition*		*Type*		*Mean of (unexposed – exposed) KHN (erosive effect)*		*SD*		*p-value*			
D Deciduous teeth		1.00 Center Fresh		49.84		34.43		0.98			
		2.00 Bubbaloo		50.29		58.37					
P Permanent teeth		1.00 Center Fresh		26.36		36.94		0.087			
		2.00 Bubbaloo		54.42		48.93					

**Table 3:** **Table 3: **Comparison of mean knoop microhardness values of unexposed and exposed areas between primary and permanent enamel to center fresh and bubbaloo (n = 15)

*Type*		*Dentition*		*Mean of (unexposed-exposed) KHN (erosive effect)*		*SD*		*p-value*			
1.00 Center Fresh		D Deciduous teeth		49.84		34.43		0.082			
		P Permanent teeth		26.36		36.94					
2.00 Bubbaloo		D Deciduous teeth		50.29		58.37		0.835			
		P Permanent teeth		54.42		48.93					

In the literature, there are studies in which cut sections were performed with diamond disk and slow speed diamond grit blades of Isomet of Buehler company.^13^ In this study cut sections were made with slow speed diamond grit blades of Isomet of Buehler Pvt. Ltd. To compare microhardness of enamel on both exposed and unexposed areas of the same tooth, cut sections of teeth through buccal window of enamel were done and microhardness was analyzed on both the surfaces simultaneously. In the literature many studies have mounted the specimens in cold cure acrylic. In this study too, cut sections of specimens were mounted in cold cure acrylic with cut section exposed for a fat surface, to facilitate microhardness study. In the previous studies knoop diamond indentations were made with 50 gm load for 10 seconds.^11^In this study too 3 knoop microhardness indentations with 50 gm load for 10 seconds were taken at both exposed and unexposed areas and mean value is calculated.

Many studies have shown that there is a significant reduction in enamel's microhardness under acidic stuffing challenge.^8,11,14^ In our study, there is significant reduction in enamel's microhardness with the exposure to whole chewing gum mixture prepared by milling the chewing gum with artificial saliva. In a study done by Bolan M, Ferreira MC, Vieira RS^8^ on erosive effects of acidic center-filled chewing gum on primary and permanent enamel, higher dental erosion is attributed to lower surface tension and higher fow. The mean knoop microhardness values of exposed area are found to be less than unexposed areas exposed to whole chewing gum milled in artificial saliva with statistically significant change or reduction in microhardness. This can be attributed to greater penetration capacity or lower surface tension of whole chewing gum mixture.^15^

In some studies, they found that erosion is different for deciduous teeth compared to permanent teeth.^14^ But, in this study, there was no statistically significant reduction in microhardness of enamel between primary and permanent enamel. In this study, the evaluation and comparison of effect of decrease in microhardness values of enamel between Center Fresh and Bubbaloo chewing gums, we found no statistically significant difference between primary and permanent enamel.

## CONCLUSION

1. There is a definite reduction in microhardness in groups exposed to whole chewing gum milled with artificial saliva.
2. Two types of chewing gums, Center Fresh and Bubbaloo are equally effective in reducing microhardness of enamel.
3. Both permanent and primary teeth are equally affected by reduction in microhardness after exposure to center filled chewing gums.
